# Effect of Forskolin on Body Weight, Glucose Metabolism and Adipocyte Size of Diet-Induced Obesity in Mice

**DOI:** 10.3390/ani11030645

**Published:** 2021-03-01

**Authors:** Jing-Yi Chen, Shao-Yu Peng, Yeong-Hsiang Cheng, I-Ta Lee, Yu-Hsiang Yu

**Affiliations:** 1 Department of Biotechnology and Animal Science, National Ilan University, Yilan 26047, Taiwan; 3311qq44@gmail.com (J.-Y.C.); yhcheng@niu.edu.tw (Y.-H.C.); 2 Department of Animal Science, National Pingtung University of Science and Technology, Pingtung 912301, Taiwan; sypeng@mail.npust.edu.tw; 3 School of Dentistry, College of Oral Medicine, Taipei Medical University, Taipei 11031, Taiwan; itlee0128@tmu.edu.tw

**Keywords:** adipocyte, forskolin, glucose, mesenchymal stem cell, mouse, obesity

## Abstract

**Simple Summary:**

Obesity has become a global pandemic involving an increase in the amount and size of fat cells in the body. Obesity is highly associated with insulin resistance and type 2 diabetes. Several medicinal plants have been reported to be useful in the prevention of obesity. Forskolin, a bioactive compound of *Coleus forskohlii*, can promote lipolysis in mature adipocytes. However, the effect of forskolin on body weight, glucose metabolism and adipocyte size of diet-induced obesity is still rarely investigated. In this study, the effects of forskolin on the high-fat diet-induced obese model were evaluated. Results showed that forskolin administration improves glucose metabolism and reduces fat cell diameter in the high-fat diet-fed mice. Forskolin also suppresses adipocyte differentiation of murine mesenchymal stem cells.

**Abstract:**

The purpose of this study was to investigate the effects of forskolin on body weight, glucose metabolism and fat cell diameter in high-fat diet-induced obese mice. Four-week-old male mice (C57BL/6) were randomly assigned to 1 of 3 treatment groups: a high-fat diet plus 5% dimethyl sulfoxide (vehicle), high-fat diet plus 2 mg/kg of forskolin (dissolved in 5% dimethyl sulfoxide) and high-fat diet plus 4 mg/kg of forskolin (dissolved in 5% dimethyl sulfoxide). Forskolin or dimethyl sulfoxide was administered intraperitoneally every two days. The results indicated that no significant difference was observed in the body weight, feed intake and serum lipid parameters among groups at 20 weeks of age. The blood glucose levels were significantly reduced in the groups treated with 2 mg/kg of forskolin before glucose tolerance test. Forskolin administration linearly decreased blood glucose levels of high-fat diet-fed mice at 90 min and total area under curve (AUC) after insulin tolerance test. The subcutaneous adipocyte diameter was significantly reduced in the groups treated with 2 mg/kg of forskolin. Forskolin administration linearly reduced the gonadal adipocyte diameter of high-fat diet-fed mice. Forskolin significantly reduced the differentiation of murine mesenchymal stem cells into adipocytes and this was accompanied by a decrease in intracellular triglyceride content and an increase in glycerol concentration in the culture medium. The subcutaneous adipocyte diameter, gonadal adipocyte diameter and total AUC of insulin tolerance test were moderately negatively correlated with the concentration of forskolin in the high-fat diet-induced obese model. These results demonstrate that forskolin can regulate glucose metabolism and reduce fat cell diameter of high-fat diet-fed mice and inhibit the adipocyte differentiation of murine mesenchymal stem cells.

## 1. Introduction

Obesity has attained the status of a global pandemic with a huge impact on human health [1]. Obesity or overweight is the leading risk factor for type 2 diabetes. Alternative strategies to reduce weight gain and prevent obesity are an urgent unmet need. The reduction in fat cell size can normalize cellular function and improve health [2]. Several anti-obesity agents, such as medicinal plants and their extracts, have been reported to have beneficial effects for the prevention of obesity [3]. These natural compounds are able to reduce fat accumulation either by attenuating adipocyte differentiation, enhancing lipolysis, or reducing lipogenesis pathways [4].

*Coleus forskohlii* is a folk medicine and has been used for treating heart diseases, respiratory disorders, constipation and intestinal disorder [5]. Forskolin, a bioactive compound of *Coleus forskohlii*, enhances adenylate cyclase enzyme activity, thereby increasing intracellular cyclic adenosine monophosphate (cAMP) concentration [6]. Increased intracellular cAMP levels elevate hormone-sensitive lipase enzyme activity by the activation of protein kinase A, resulting in promoting lipolysis in mature adipocytes [7,8,9]. In addition to lipolysis, cAMP also involves the regulation of adipocyte differentiation. It has been reported that forskolin promotes the differentiation of murine primary adipocyte precursor and pre-adipocyte cell line [10,11]. However, forskolin treatment is unable to trigger the adipocyte differentiation of swine stromal-vascular fraction [12]. Thus, the effect of forskolin on the regulation of adipocyte differentiation still remains yet to be elucidated.

It has been demonstrated that *Coleus forskohlii* extract supplementation does not alter the body weight of mice without affecting feed intake under a normal-fat diet feeding [13,14]. A significant reduction of visceral fat weight is observed in the *Coleus forskohlii* extract-fed mice [13]. The increased blood triglyceride levels and fatty liver are found in mice after a high dosage of *Coleus forskohlii* extract supplementation under a normal-fat diet feeding [14]. However, it has been demonstrated that *Coleus forskohlii* extract supplementation does not alter the body weight of mice under high-fat feeding conditions unless given in high doses which also decrease food intake [13,15]. Subcutaneous and visceral adipocyte size is an important indicator of insulin resistance and highly correlates with glucose metabolism [16,17]. Adipocyte hypertrophy in adipose tissue impairs glucose metabolism and insulin sensitivity in humans [2]. Previous studies mainly focus on the effects of *Coleus forskohlii* extract or forskolin on body weight and fat weight under normal-fat or high-fat diet feeding [13,14,15]. It is particularly important to demonstrate whether forskolin can reduce the fat cell diameter and normalize glucose metabolism in the diet-induced obesity model.

Therefore, this study aimed to investigate the effects of forskolin on glucose and insulin tolerance as well as adipocyte diameter in diet-induced obesity of mice. The results provide a theoretical basis for the use of forskolin as a functional food for the prevention of obesity and type 2 diabetes.

## 2. Materials and Methods

The National Ilan University Institutional Animal Care and Use Committee (IACUC protocol 105-48) approved all animal procedures, which adhered to the ethical and humane use of animals for research.

### 2.1. Animals and Diets

Male C57BL/6 mice at 4 weeks of age were randomly allocated to three different treatment groups in a completely randomized design (n = 8–11 per group): (1) high-fat diet group (60% calorie from fat, TestDiet 58Y1), (2) high-fat diet group plus intraperitoneal administration of forskolin (2 mg/kg) and (3) high-fat diet group plus intraperitoneal administration of forskolin (4 mg/kg). All mice were housed at 23 °C and light/dark cycles of 12/12 h. Mice were fed ad libitum with a high-fat diet. Forskolin was purchased from Sigma-Aldrich (F6886, St. Louis, MO, USA). Forskolin (dissolved in 5% dimethyl sulfoxide) was administered intraperitoneally at a concentration of 2 or 4 mg/kg every two days. For the high-fat diet alone group, dimethyl sulfoxide was administered intraperitoneally at a concentration of 5% every two days. The experimental period was 16 weeks.

### 2.2. Histology

Three mice per group were chosen at the end of the experiment (20 weeks old) based on their average weight being within 5% of their average body weight and sacrificed by cervical dislocation with anesthesia (tribromoethanol, 0.4 mg/g of body weight, intraperitoneal injection; Sigma, St. Louis, MO, USA). White adipose tissue was collected and fixed in 4% paraformaldehyde and embedded in paraffin. Sections were cut and stained with hematoxylin/eosin staining. For adipocyte size measurements, 200 consecutive fat cells of the subcutaneous and gonadal fat pad from each mouse were selected for the area measurement using Image J software (version 1.44, http://rsbwed.nih.gov/ij/ (accessed on 30 December 2020)).

### 2.3. Glucose Tolerance Test and Insulin Tolerance Test

For glucose tolerance test, eight mice per group (20 weeks old) were fasted overnight, blood samples were collected from tail vein and glucose concentrations were measured at 0 min and 30 min and 90 min after intraperitoneal injection of glucose (1 g/kg body weight). For insulin tolerance test, eight mice per group (20 weeks old) were fasted for 6 h, blood samples were collected from tail vein and glucose concentrations were measured at 0 min and 30 min and 90 min after intraperitoneal injection of bovine insulin (I0516, Sigma-Aldrich, St. Louis, MO, USA) at 0.75 units/kg body weight. Blood glucose was measured using a Accu-Chek glucometer (Roche Diagnostics, Mannheim, Germany).

### 2.4. Cell Culture and Mesenchymal Stem Cell Differentiation

C3H10T1/2 mesenchymal stem cells (CCL-226, American Type Culture Collection, Manassas, USA) were cultured in Dulbecco’s modified Eagle medium (DMEM, Sigma-Aldrich, St. Louis, MO, USA) with 10% fetal bovine serum (FBS, Thermo Fisher Scientific, Waltham, MA, USA) at 37 °C in an atmosphere of 5% CO2. To determine the optimal concentration of forskolin for using in this study, dose-dependent cytotoxicity was examined with different concentrations of forskolin (0–60 μM). Briefly, 1 × 10^3^ cells were seeded into each well of 96-well microtiter plates and treated with the indicated concentrations of forskolin. After 48 h of treatment, the percentage of viable cells was quantified by measuring the absorbance at 490 nm using a microtiter culture plate reader (MTS Assay Kit, Abcam, Cambridge, MA, USA). For terminal adipocyte differentiation, confluent C3H10T1/2 mesenchymal stem cells were cultured in the induction medium (DMEM containing 10% FBS, 1 μM dexamethasone, 0.5 mM 3-isobutyl-1-methylxanthine and 5 μg/mL insulin) with different concentrations of forskolin for 2 days. The cells were then cultured in growth medium (DMEM containing 10% FBS) and different concentrations of forskolin (0–10 μM) for 6 days, with a medium change every 2 days. The dexamethasone, 3-isobutyl-1-methylxanthine and insulin were purchased from Sigma-Aldrich (St. Louis, MO, USA). At the end of the experiment, the cells were stained in the culture plates with Oil-Red O reagent (Sigma-Aldrich, St. Louis, MO, USA) to measure the degree of lipid accumulation. The culture medium was harvested for glycerol measurement. Experiments were performed with three independent experiments (performed at least in duplicate on different cell passages and different dates, each consists of three replicates per treatment).

### 2.5. Glycerol, Free Fatty Acid and Triglyceride Level Analysis

At the end of the experiment (20 weeks old), blood samples from eight mice per group were collected from the facial vein and separated after centrifugation at 1500 *g* for 10 min. The concentrations of glycerol, free fatty acid and triglyceride in the serum were measured using commercial assay kits (10011725, Cayman Chemical, Ann Arbor, MI, USA; K612-100, Biovision, Milpitas, CA, USA; K622-100, Biovision, Milpitas, CA, USA) according to the manufacturers’ instructions. The concentration of glycerol in the culture medium was measured using commercial assay kits (10011725, Cayman Chemical, Ann Arbor, MI, USA) according to the manufacturers’ instructions.

### 2.6. Quantitative Reverse Transcription-PCR

Total RNA was reverse transcribed into complementary DNA using a Transcriptor Reverse Transcriptase kit (Roche Applied Science, Indianapolis, IN, USA). Quantitative reverse transcription-PCR was performed using a MiniopticonTM Real-Time PCR Detection System (Bio-Rad, Hercules, CA, USA) and KAPA SYBR FAST qPCR Kit (Kapa Biosystems, Inc., Boston, MA, USA). The internal control gene was 18S rRNA. The primers are as follows: glucose transporter 4 (*glut4*) forward: 5′-ACATACCTGACAGGGCAAGG′-3′ and reverse: 5′-CGCCCTTAGTTGGTCAGAAG′-3′; 18S rRNA (*18S*) forward: 5′-ACGATGCCGACTGGCGATGC-3′ and reverse: 5′- TCCTGGTGGTGCCCTTCCGT-3′. mRNA expression of each gene was normalized to the 18S gene in the same sample. Threshold cycle (Ct) values were obtained and the relative gene expression was calculated using the formula 2^−ΔΔCt^.

### 2.7. Statistical Analysis

All data were analyzed by one-way ANOVA through the general linear model procedure of SAS (SAS Institute, Cary, USA). Means were compared using Tukey honestly significant difference test at a significance level of *p* ≤ 0.05. The linear-quadratic (LQ) dose-effect was used to investigate the dose response and biologically effective dose of forskolin in mice. The relationship between forskolin concentration, body weight, serum lipid parameters (triglyceride, free fatty acid and glycerol), adipocyte diameter (subcutaneous and gonadal fat) and total area under curve (glucose tolerance test and insulin tolerance test) in the same mice of different groups was analyzed by Pearson’s correlation coefficient (r).

## 3. Results

### 3.1. Effects of Forskolin on Body Weight, Serum Lipids and Glucose Metabolism of High-Fat Diet-Fed Mice

**Figure 1:**
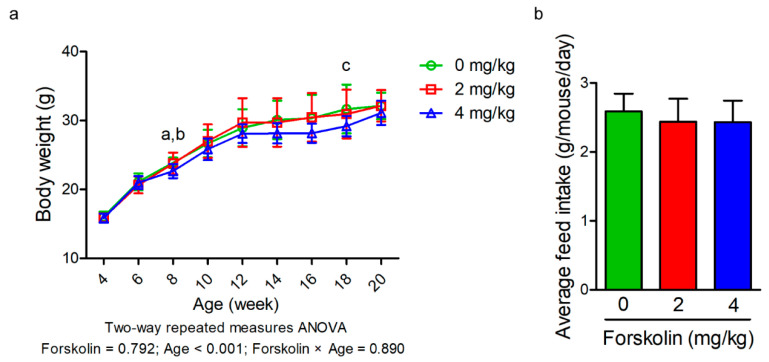
Effects of forskolin on body weight and feed intake of diet-induced obesity in mice. (**a**) Body weight of high-fat diet-fed mice in response to forskolin administration. Data are average body weight (g/mouse) of 8–11 mice per treatment. ^a^ *p* value = 0.067 by one-way ANOVA. ^b^ *p* value = 0.049 by linear contrasts. ^c^ *p* value = 0.096 by linear contrasts. (**b**) Average feed intake of high-fat diet-fed mice from the age of 10 to 16 weeks in response to forskolin administration. Data are average feed intake (g/mouse/day) of 8–11 mice per treatment.

**Figure 2:**
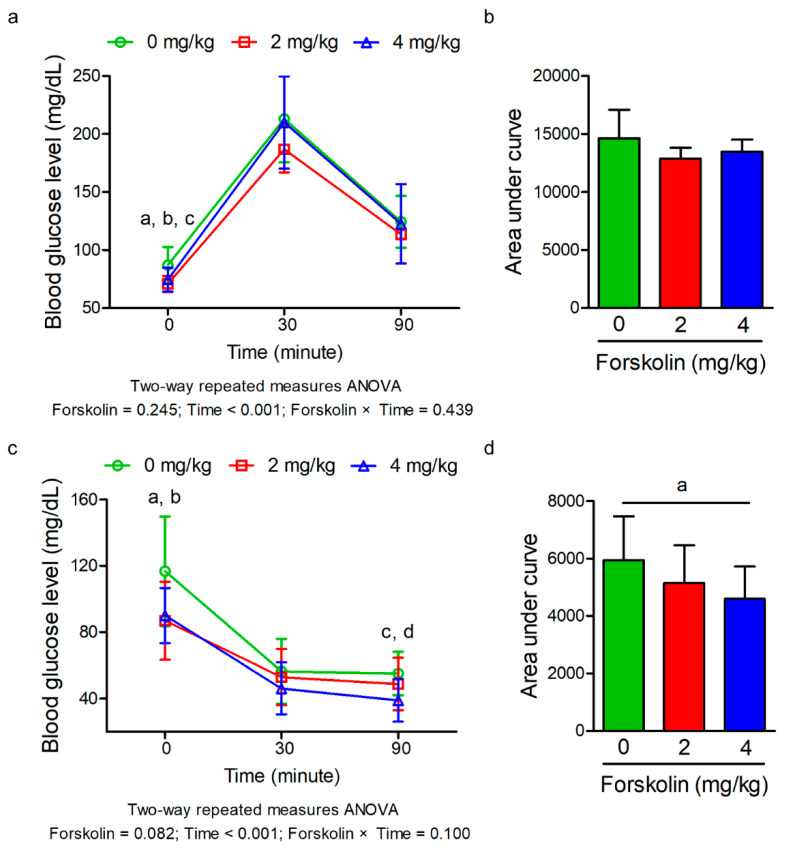
Effect of forskolin on glucose metabolism of high-fat diet-induced obesity in mice. (**a**) Blood glucose levels of high-fat diet-fed mice in response to glucose tolerance test. Data are average blood glucose levels of 8 mice per treatment. ^a^ *p* value = 0.025 by one-way ANOVA. ^b^ *p* value = 0.054 by linear contrasts. ^c^ *p* value = 0.057 by quadratic contrasts. (**b**) Area under curve of high-fat diet-fed mice in response to glucose tolerance test. Data are average blood glucose levels of 8 mice per treatment. The bars indicate mean ± SD. (**c**) Blood glucose levels of high-fat diet-fed mice in response to insulin tolerance test. Data are average blood glucose levels of 8 mice per treatment. ^a^ *p* value = 0.053 by one-way ANOVA. ^b^ *p* value = 0.052 by linear contrasts. ^c^ *p* value = 0.090 by one-way ANOVA. ^d^ *p* value = 0.028 by linear contrasts. (**d**) Area under curve of high-fat diet-fed mice in response to insulin tolerance test. Data are average blood glucose levels of 8 mice per treatment. The bars indicate mean ± SD. ^a^ *p* value = 0.050 by linear contrasts.

The effect of forskolin on body weight of mice under diet-induced obesity is shown in Figure 1a. The results revealed that no significant difference in body weight was found during the experimental period among the groups. A trend of decreased body weight was observed with the administration of forskolin at 8 weeks of age (*p* = 0.067, one-way ANOVA). A linear reduction in body weight of high-fat diet-fed mice was found as the inclusion level of forskolin increased at 8 and 18 weeks of age (*p* ≤ 0.05 and *p* = 0.096). No significant difference in feed intake was found among three groups (Figure 1b). Forskolin administration did not cause a significant effect on serum lipid parameters of high-fat diet-fed mice (Table 1). A quadratic trend in serum free fatty acid levels of high-fat diet-fed mice was observed as the inclusion level of forskolin increased (*p* = 0.094). The effect of forskolin on glucose metabolism of diet-induced obesity in mice is shown in Figure 2. In the glucose tolerance test, the blood glucose levels were significantly reduced in the groups treated with 2 mg/kg of forskolin before the administration of glucose (*p* < 0.05, one-way ANOVA) (Figure 2a). No significant difference in glucose tolerance test was found among the groups (Figure 2a). A linear and quadratic trend in blood glucose levels of high-fat diet-fed mice was observed as the inclusion level of forskolin increased before glucose administration (*p* = 0.054 and *p* = 0.057) (Figure 2a). There were no significant differences in blood glucose levels and total area under curve (AUC) among three groups after the administration of glucose (Figure 2b). In the insulin tolerance test, a trend of decreased blood glucose levels of high-fat diet-fed mice was observed with the administration of forskolin at 0 and 90 min after intraperitoneal injection of insulin (*p* = 0.053 and *p* = 0.090) (Figure 2c). No significant difference in insulin tolerance test was found among the groups (Figure 2c). A linear trend in blood glucose levels of high-fat diet-fed mice was observed as the inclusion level of forskolin increased before intraperitoneal injection of insulin (*p* = 0.052) (Figure 2c). Forskolin administration linearly decreased blood glucose levels of high-fat diet-fed mice at 90 min after intraperitoneal injection of insulin (*p* ≤ 0.05) (Figure 2c). A linear response in the total AUC of high-fat diet-fed mice was also observed as the inclusion level of forskolin increased after insulin tolerance test (*p* ≤ 0.05) (Figure 2d).

### 3.2. Effects of Forskolin on Fat Cell Diameter of High-Fat Diet-Fed Mice and Adipocyte Differentiation of Mesenchymal Stem Cells

**Figure 3:**
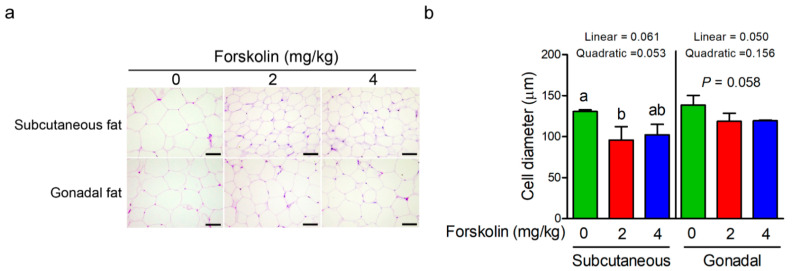
Effect of forskolin on adipocytes of high-fat diet-induced obesity in mice. (**a**) Hematoxylin and eosin staining of the subcutaneous and gonadal fat tissue of mice fed on high-fat diet in response to forskolin treatment. Three mice samples per group (n = 3) were stained and one representative result is shown. Bars indicate a length of 100 μm. (**b**) Effect of forskolin on the fat cell diameter of diet-induced obesity in mice. Subcutaneous and gonadal adipose section from three mice samples per group (n = 3) were measured. The bars indicate mean ± SD. ^a,b^ Means with no common superscript are significantly different (*p* ≤ 0.05).

**Figure 4:**
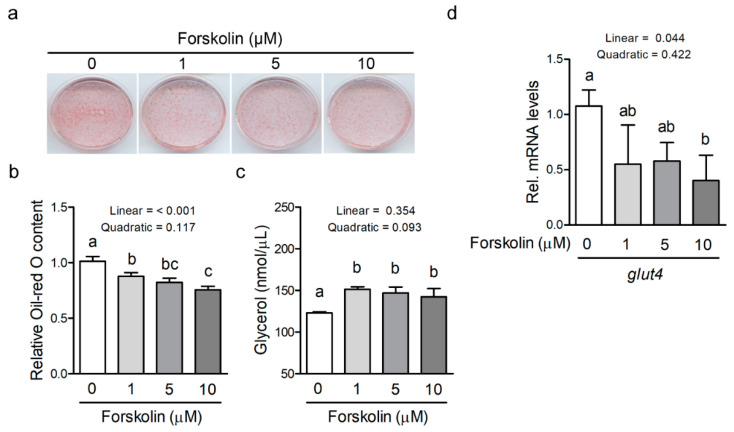
Effect of forskolin on adipocyte differentiation. (**a**) Oil-Red O staining of forskolin-treated mesenchymal stem cells after stimulation using an adipogenic induction cocktail for 8 days to promote differentiation. Three experiments (n = 3) were carried out and one representative result is shown. (**b**) Relative triglyceride content quantified via Oil Red O staining in forskolin-treated mesenchymal stem cells after stimulation using an adipogenic induction cocktail for 8 days. Three experiments (n = 3) were carried out. The bars indicate mean ± SD. ^a–c^ Means with no common superscript are significantly different (*p* ≤ 0.05). (**c**) glycerol levels of culture medium in forskolin-treated mesenchymal stem cells after stimulation using an adipogenic induction cocktail for 8 days. Three experiments (n = 3) were carried out. The bars indicate mean ± SD. ^a,b^ Means with no common superscript are significantly different (*p* ≤ 0.05). (**d**) mRNA expression of glucose transporter 4 (glut4) in mesenchymal stem cells treated with the adipogenic induction cocktail and forskolin for 8 days (n = 3). The bars indicate mean ± SD. ^a,b^ Means with no common superscript are significantly different (*p* ≤ 0.05).

The effect of forskolin on fat cell diameter of diet-induced obesity in mice is shown in Figure 3. The subcutaneous and gonadal adipocyte diameter of forskolin-treated mice was smaller than those of control mice (Figure 3a). After quantification of fat cell diameter, the subcutaneous adipocyte diameter was significantly reduced in the group treated with 2 mg/kg of forskolin (*p* ≤ 0.05) compared with 0 mg/kg of forskolin (Figure 3b). A linear and quadratic trend in the subcutaneous adipocyte diameter of high-fat diet-fed mice was observed as the inclusion level of forskolin increased (*p* = 0.061 and *p* = 0.053) (Figure 3b). A trend of decreased gonadal adipocyte diameter of high-fat diet-fed mice was found with the administration of forskolin (*p* = 0.058) (Figure 3b). Forskolin administration linearly reduced the gonadal adipocyte diameter of high-fat diet-fed mice (*p* ≤ 0.05) (Figure 3b). To determine whether forskolin can regulate adipocyte differentiation, murine mesenchymal stem cells were treated with forskolin and were then induced for adipocyte differentiation. The result of cytotoxicity showed that 60 µM of forskolin significantly affected the proliferation of mesenchymal stem cells (*p* ≤ 0.05), whereas 10 µM of forskolin did not impair the proliferation of mesenchymal stem cells (Table 2). Thus, forskolin concentration below 10 µM was selected for subsequent experiments. After 8-day adipogenic induction, adipocyte differentiation dose-dependently decreased in forskolin-treated cells as compared with control cells (Figure 4a). The intracellular triglyceride content quantified via Oil Red O staining was significantly reduced in the groups treated with forskolin (Figure 4b, *p* ≤ 0.05). A linear response in the intracellular triglyceride content was observed as the inclusion level of forskolin increased (Figure 4b, *p* ≤ 0.05). The glycerol concentration in the culture medium was significantly increased in the groups treated with forskolin after 8-day adipogenic induction (Figure 4c, *p* ≤ 0.05). A quadratic trend in the glycerol concentration in the culture medium was observed as the inclusion level of forskolin increased (Figure 4c, *p* = 0.093). The expression of glut4 gene decreased in 10 μM forskolin-treated cells compared with control cells (*p* ≤ 0.05) (Figure 4d). A linear reduction in glut4 mRNA levels was also observed as the inclusion level of forskolin increased (*p* ≤ 0.05) (Figure 4d).

### 3.3. Association between Forskolin Concentration, Body Weight, Serum Lipid Parameters, Fat Cell Diameter and Total AUC of Glucose and Insulin Tolerance Test

**Figure 5:**
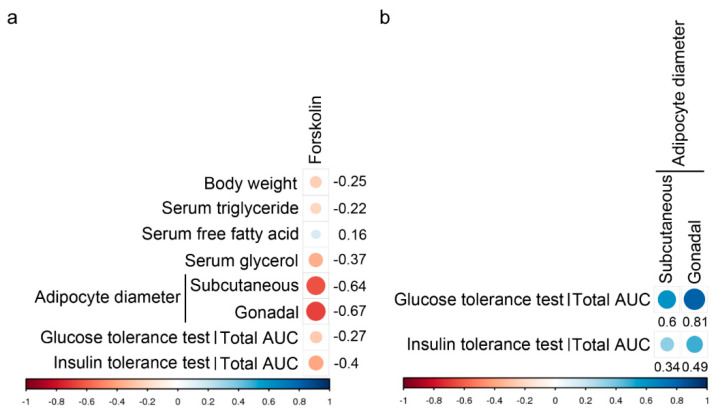
Pearson’s correlation analysis. (**a**) The correlation coefficient between the concentration of forskolin with body weight, serum lipid parameters (triglyceride, free fatty acid and glycerol), adipocyte size (subcutaneous and gonadal fat), or total AUC (glucose tolerance test and insulin tolerance test) in high-fat diet-induced mice. (**b**) The correlation coefficient between adipocyte size (subcutaneous and gonadal fat) and total AUC (glucose tolerance test and insulin tolerance test) in high-fat diet-induced mice. Positive correlations are displayed in blue and negative correlations are in red color. Circle sizes are proportional to the correlation coefficients.

The results of correlation analysis between forskolin concentration, body weight, serum lipid parameters, fat cell diameter and total AUC in the high-fat diet-fed mice of different groups are shown in Figure 5a. The body weight (r = −0.25), serum triglyceride levels (r = −0.22), serum glycerol levels (r = −0.37) and total AUC of glucose tolerance test (r = −0.27) were slightly negatively correlated with the concentration of forskolin. The subcutaneous adipocyte diameter (r = −0.64), gonadal adipocyte diameter (r = −0.67) and total AUC of insulin tolerance test (r = −0.4) were moderately negatively associated with the concentration of forskolin. However, the serum free fatty acid levels (r = 0.16) were slightly positively correlated with the concentration of forskolin. The results of correlation analysis between the fat cell diameter and total AUC in the high-fat diet-fed mice of different groups are shown in Figure 5b. The subcutaneous adipocyte diameter (r = 0.6) was moderately positively associated with the total AUC of glucose tolerance test. The gonadal adipocyte diameter (r = 0.81) was strongly positively associated with the total AUC of glucose tolerance test. The subcutaneous adipocyte diameter (r = 0.34) was slightly positively associated with the total AUC of insulin tolerance test. The gonadal adipocyte diameter (r = 0.49) was moderately positively associated with the total AUC of insulin tolerance test.

## 4. Discussion

In this study, we demonstrated for the first time that forskolin administration decreased blood glucose levels of high-fat diet-fed mice at 90 min and total AUC after intraperitoneal injection of insulin. The subcutaneous and gonadal adipocyte diameter of high-fat diet-fed mice was reduced in response to forskolin administration. Forskolin attenuated the adipocyte differentiation of murine mesenchymal stem cells and this was accompanied by a decrease in intracellular triglyceride levels and an increase in glycerol concentration in the culture medium. The concentration of forskolin was moderately negatively associated with subcutaneous adipocyte diameter, gonadal adipocyte diameter and total AUC of insulin tolerance test.

In humans, it has been reported that oral ingestion of forskolin causes a significant decrease in fat percentage and fat mass in overweight and obese men [18]. In another study, the body weight and fat mass in mildly overweight women are not improved after *Coleus forskohlii* extract supplementation [19]. *Coleus forskohlii* extract or forskolin supplementation does not alter the body weight of mice under a normal-fat diet, whereas the visceral fat weight of *Coleus forskohlii* extract or forskolin-fed mice is reduced [13,14]. *Coleus forskohlii* extract supplementation does not affect the serum cholesterol, phospholipid and free fatty acid levels in normal-fat diet-fed mice, except blood triglyceride levels [14]. In contrast, the serum free fatty acid levels are increased in high-fat diet-fed mice after forskolin administration [15]. In the present study, we found that forskolin administration did not cause a significant effect on serum lipid parameters of high-fat diet-fed mice. It has been demonstrated that orogastric administration of forskolin extract in combination with high-fat diet feeding does not alter the body weight of mice [15]. Similarly, the body weight of high-fat diet-fed mice was not affected after forskolin administration in the present study. However, we demonstrated that subcutaneous and gonadal adipocyte diameter of high-fat diet-fed mice was reduced in response to forskolin administration. The regulation of adipocyte differentiation and lipolysis in differentiated adipocytes can control the adipocyte size [2]. It has been demonstrated that forskolin is able to promote lipolysis in mature adipocytes by the activation of hormone-sensitive lipase enzyme activity [7,8,9]. Here, we further demonstrated forskolin could attenuate the adipocyte differentiation of murine mesenchymal stem cells. Thus, forskolin administration may regulate adipocyte differentiation and lipolysis in the adipose tissue of high-fat diet-fed mice, resulting in the reduction of fat cell diameter. However, the precise mechanisms of lipid metabolism mediated by forskolin in the adipose tissue in response to high-fat diet feeding should be investigated in future studies. In addition, whether forskolin administration can reshape the body composition (fat mass and lean mass) and normalize adipocyte hypertrophy-associated pathways (insulin resistance and chronic inflammation) is still needed to be elucidated. Taken together, these findings demonstrate that *Coleus forskohlii* extract and forskolin supplementation can reduce the fat mass and adipocyte size in human and rodent models.

Overweight and obesity are risk factors for developing insulin resistance and type 2 diabetes [20]. Hepatic steatosis is highly associated with insulin resistance [21]. It has been reported that *Coleus forskohlii* extract supplementation induces hepatic steatosis in normal-fat diet-fed mice [13,14,22]. Whether *Coleus forskohlii* extract-induced fatty liver impairs glucose metabolism in mice is still unclear. In contrast, forskolin administration does not cause hepatotoxicity in mice [13]. In the present study, the fasting blood glucose levels were reduced in the group treated with 2 mg/kg of forskolin. In addition, forskolin administration also decreased blood glucose levels of high-fat diet-fed mice after intraperitoneal injection of insulin, indicating that forskolin could improve insulin sensitivity. Previous studies have demonstrated that insulin sensitivity is inversely correlated with subcutaneous and visceral adipocyte size in humans [2,16], indicating that reduction of fat cell diameter by inhibiting adipocyte differentiation or enhancing lipolysis can mitigate insulin sensitivity. Here, the subcutaneous and gonadal adipocyte diameter of high-fat diet-fed mice in combination with forskolin administration was positively correlated with total AUC. The reduced subcutaneous and visceral adipocyte size may normalize the insulin signaling transduction of adipocytes and these effects are mainly through forskolin-mediated lipolysis in hypertrophic adipocytes. In addition, forskolin treatment reduces adipocyte differentiation of murine mesenchymal stem cells. Thus, inhibition of adipocyte differentiation and promotion of lipolysis by forskolin administration in adipose tissue might contribute to the reduction of fat cell size, thereby improving glucose metabolism. In addition, to adipose tissue, whether forskolin also involves in the regulation of pancreatic β cell function or insulin secretion still needs to be verified in the future. Overall, the benefits of forskolin on weight loss and reduced adipose cell size through inhibiting adipocyte differentiation of mesenchymal stem cells or promoting lipolysis in mature adipocytes may normalize insulin sensitivity under high-fat diet feeding, thereby exhibiting a preventive effect on type 2 diabetes.

The effects of forskolin on lipolysis by increased cellular cAMP concentration and activation of hormone-sensitive lipase enzyme activity in differentiated adipocytes have been well-studied [7,8,9]. The elevation of cellular cAMP concentration also positively regulates the early program of differentiation [23,24]. It has been demonstrated that forskolin promotes the differentiation of murine primary adipocyte precursor and pre-adipocyte cell line [10,11]. Here, we demonstrated that forskolin significantly reduced the differentiation of murine mesenchymal stem cells into adipocytes. The previous study indicates that a strong and sustained increase in cAMP levels can inhibit the process of adipogenesis [25]. Since the mesenchymal stem cells were simultaneously treated with forskolin and 3-isobutyl-1-methylxanthine (a cAMP activator) during early adipocyte differentiation in the present study, the intracellular cAMP concentration may reach the inhibitory concentration of adipogenesis upon forskolin and 3-isobutyl-1-methylxanthine treatment. The actual intracellular cAMP concentration during adipocyte differentiation of murine mesenchymal stem cells in response to forskolin treatment still needs to be measured. Taken together, these findings indicate that forskolin is able to regulate murine adipocyte differentiation and these effects may depend on the intracellular cAMP concentration during the early stages of adipocyte differentiation.

Regarding the effective dose of forskolin in mice, only one study reported that forskolin does not alter the body weight of mice under a high-fat diet feeding [15]. Similarly, we also found that forskolin did not change the body weight of mice under a high-fat diet feeding even the forskolin concentration was increased (4 mg/kg of body weight) as compared with a previous study (0.5 mg/kg of body weight) [15]. Under normal-fat diet feeding, forskolin with a concentration range from 0.005 to 0.5 mg/g of feed does not has an impact on body weight of mice [13,14,22]. When the dose is increased to 5 mg/g of feed, the body weight was significantly reduced [13,22]. Based on these findings, 5 mg forskolin/g of feed is high enough to reduce body weight in the mouse model. However, it is unclear whether the dosage is still able to reduce body weight in high-fat diet-induced obese model.

Long-term excessive energy intake disrupts the energy metabolism and adipocytes are induced to become hypertrophic and insulin resistant. Here, we found that forskolin administration decreased the size of hypertrophic adipocytes in adipose tissues without altering the body weight and feed intake, implying energy partitioning among organs was regulated in forskolin-treated high-fat diet-fed mice. Although it has been demonstrated that *Coleus forskohlii* extract or forskolin can reduce the fat mass in obese humans and mice [13,14,18]. It is particularly important to investigate the effect of forskolin on energy partitioning among organs, such as adipose tissue and skeletal muscle in the future. Adipocyte size plays an important role in energy and glucose metabolism and can be regulated by adipocyte differentiation, lipolysis and lipogenesis. The lipolytic potential of forskolin has been widely demonstrated in the past years in the in vitro models. Here, we demonstrated that forskolin exerts an anti-adipogenic effect on adipocyte differentiation of murine mesenchymal stem cells. Although the effect of forskolin on lipogenesis pathways has not been confirmed, we may reasonably hypothesize that forskolin potentially reduces fat cell size of the high-fat diet-fed mice by increasing lipolysis, attenuating adipocyte differentiation, or both. Since glucose metabolism is improved after forskolin administration in the present study, many questions are left unanswered. For instance, whether the insulin signaling pathway and inflammatory response in adipose tissue are improved by forskolin still needs to be elucidated.

## 5. Conclusions

Forskolin administration improves glucose metabolism and reduces fat cell diameter in the high-fat diet-fed mice. Forskolin negatively regulates adipocyte differentiation of murine mesenchymal stem cells. Further research should investigate whether forskolin stimulates interorgan crosstalk between the adipose tissue, liver and skeletal muscle to contribute to lipid and glucose metabolism.

## Figures and Tables

**Table 1:** Effect of forskolin on serum lipid profile of diet-induced obesity in mice.

Item	Forskolin (mg/kg)			SEM ^2^	*p* Value	*p* Value	
	0	2	4			Linear	Quadratic
Triglyceride (nmol/μL)	0.06 ^1^	0.04	0.05	0.006	0.372	0.380	0.275
Free fatty acid (nmol/μL)	0.76	0.61	0.87	0.057	0.181	0.454	0.094
Glycerol (nmol/μL)	14.6	12.7	11.8	0.730	0.307	0.126	0.763

^1^ Data are mean values of 8 mice per treatment; ^2^ SEM = standard error of mean.

**Table 2:** Dose-dependent cytotoxicity of forskolin on C3H10T1/2 mesenchymal stem cells.

Forskolin (μM)	Relative Cell Number
0	1.16 ^a,1^
10	0.89 ^ab^
20	0.87 ^ab^
30	0.91 ^ab^
40	0.88 ^ab^
50	0.85 ^ab^
60	0.72 ^b^
SEM ^2^	0.038
*p* value	0.019

^1^ Data are mean values of 3 replicates per treatment; ^2^ SEM = standard error of mean; ^a,b^ Means of a column with no common superscript are significantly different (*p* ≤ 0.05).

## Data Availability

The data presented in this study are available on reasonable request from the corresponding author.
